# Efficacy and safety of low-concentration whitestrips for extrinsic tooth discoloration: a randomized controlled trial

**DOI:** 10.3389/froh.2026.1729993

**Published:** 2026-04-14

**Authors:** Yuwei Wang, Di Fu, Lin Yao, Yisi Zhong, Ling Zou, Jing He, Wei Yin

**Affiliations:** State Key Laboratory of Oral Diseases & National Center for Stomatology & National Clinical Research Center for Oral Diseases & West China Hospital of Stomatology, Sichuan University, Chengdu, China

**Keywords:** hydrogen peroxide, randomized controlled trial, safety, tooth whitening, tooth discoloration

## Abstract

**Objectives:**

This study aimed to evaluate the effectiveness of Low-concentration Whitestrips with 3% hydrogen peroxide (HP) on extrinsic discoloration of incisors, and to evaluate the safety of whitening process.

**Methods:**

Enrolled adults with extrinsic discolored incisors (Vita shade guide value >1M1.5) were randomized into treatment and control groups. Treatment group used Low-concentration Whitestrips, control group used placebo. Tooth color was assessed at baseline and follow-up visits at Day 1, Day 3 and Day 14. Tooth color was assessed by digital shade meter (primary outcomes), and VITA 3D-Master shade guide (secondary outcomes), respectively. Adverse events (AEs) and serious adverse events (SAEs) including oral and systemic symptoms based on clinical examination and participants’ description were also recorded.

**Results:**

70 adults participated. The primary and secondary outcomes objectively and subjectively demonstrated that the treatment group exhibited better whitening effects than the control. Additionally, the treatment group was observed to attain better effects with prolonged use, with W* values based on digital shade meter increasing from 72.86 to 78.73, and the VITA 3D-Master shade guide color values decreasing from 14.91 to 11.23 (*P* < 0.05). Five AEs occurred with no significant difference between groups (*P* > 0.05). No SAEs were reported.

**Conclusions:**

From both objective and subjective perspectives, Low-concentration Whitestrips were found to be effective in extrinsic discoloration tooth bleaching and were well-tolerated by participants.

**Clinical Trial Registration:**

Chinese Clinical Trial Registry: https://www.chictr.org.cn/ (ChiCTR2300073971) and National Health Information Platform: http://114.255.48.20/login (MR-51-23-033324).

## Introduction

Extrinsic tooth discoloration refers to the phenomenon where teeth exhibit a change in coloration due to the external accumulation of pigments from substances such as food, beverages, and tobacco on the enamel's surface or within its composition (1). As people's demand for an aesthetically pleasing smile has increased, there has been a sharp rise in the demand for tooth whitening worldwide. It has been recognized that the localized use of hydrogen peroxide (HP) for bleaching teeth has been in practice since the 1980s. Now the effectiveness of various whitening strips with HP as the active ingredient have been proven and they are widely used in clinical whitening (2).

HP is a highly permeable small molecule that can easily penetrate the enamel surface to the deeper layers of the tooth, breaking down large pigment molecules into smaller ones. These smaller molecules spread to the surface of the tooth and are absorbed, resulting in a whitening effect (3). High-concentration HP (25%–40%) tooth bleaching products are commonly used in in-office bleaching (4), and physical barriers are required for hard tissue prophylaxis and soft tissue protection. However, high-concentration HP has been reported to have multiple risks. The sensitivity of teeth during and after bleaching is related to microscopic surface defects and enamel pores that allow the bleaching agents to enter the pulp quickly. High-concentration HP can also induce inflammatory responses and lead to necrosis of the pulp tissue due to its irritating and cytotoxic potential (5). Gingival irritation and corrosion of mucous membranes and skin have also been reported (6).

At-home bleaching requires bleaching products with lower concentrations, one of which is tray bleaching containing 10%–20% carbamide peroxide (CP) (7, 8). Another option is whitening strips, which can avoid the step of customizing the tray compared to tray bleaching, thus being more convenient. Whitening strips at-home bleaching typically contain 5%–15% HP, this kind of low-concentration HP is considered gentler and is also effective for removing extrinsic tooth discoloration (9), but still presents a light cytotoxicity (10–13). Therefore, in order to pursue a more safe and comfortable whitening effect, Low-concentration Whitestrips used in this study only contain 3% HP.

For a comprehensive assessment of tooth color change when performing bleaching with Low-concentration Whitestrips, the Vita Easyshade digital shade guide was used to assess the primary outcomes, providing an objective evaluation of tooth color, while the secondary outcomes were determined using the VITA 3D-Master shade guide for a subjective evaluation. Both methods were used to evaluate the same clinical outcome, namely tooth color change, allowing complementary assessment from instrumental and visual perspectives. At the same time, adverse events (AEs) and serious adverse events (SAEs) were recorded during the process.

Therefore, a prospective, single-center, parallel, controlled, randomized, double-blind clinical trial was conducted to evaluate the effectiveness and safety of the Low-concentration Whitestrips. We tested the following three null hypotheses: that bleaching with low-concentration Whitestrips (1) does not result in significant tooth color change, and (2) does not influence the incidence of AEs/SAEs.

## Materials and methods

### Ethical aspects

All procedures were performed in compliance with relevant laws and institutional guidelines and have been approved by the appropriate institutional committees. This study was approved by the Ethics Committee of West China Hospital of Stomatology Sichuan University for the protection of human participants (WCHSIRB-D-2023-199). It was also registered in National Health Information Platform (MR-51-23-033324) and Chinese Clinical Trial Registry (ChiCTR2300073971). And the privacy rights of human subjects have been observed and that informed consent was obtained for experimentation with human subjects. We used the CONSORT checklist when writing our report (14).

### Sample size

This is an exploratory study, and therefore, a sample size calculation based on statistical power was not feasible due to the absence of prior data. Instead, the sample size was justified based on similar clinical studies and the guidelines from the China Oral Care Industry Association (T/COCIA 8-2020). A target of 60 evaluable subjects was set. To compensate for an anticipated dropout rate of 15%, approximately 70 participants will be enrolled in the study.

### Recruitment

Evaluations were conducted during the clinical process. The eligibility criteria are as follows:

Inclusion Criteria

1. Age between 18 and 55 years, with no gender restrictions;
2. In good general health, without serious systemic diseases;
3. Eight incisors should be naturally well-aligned and without restorations or prosthetic crowns;
4. Have extrinsic tooth discoloration with maxillary incisors Vita shade guide value >1M1.5 evaluated by professionally trained dentists;
5. Willingly participate in the trial, comply with clinical trial requirements, and follow up on time.

Exclusion Criteria

1. Untreated dental caries, mucosal diseases, dentin sensitivity, periodontitis, or other diseases in the teeth to be examined;
2. Crown restorations, dentures, or orthodontic appliances on the teeth to be examined;
3. Extensive restorations, suspected pulpitis, caries, enamel cracks, etc., in the teeth to be examined;
4. Severe or atypical intrinsic tooth discoloration;
5. Individuals with allergies (mainly referring to those allergic to HP and other ingredients of the product);
6. Women who are pregnant or breastfeeding;
7. Use of other tooth whitening products within the past 3 months;
8. Currently taking anticonvulsants, antihistamines, antidepressants, sedatives, tranquilizers, anti-inflammatory drugs, daily painkillers, etc.;
9. Patients with serious primary diseases of the cardiovascular, liver, kidney, and hematopoietic systems, as well as diseases of the nervous and mental systems;
10. Patients with oral soft and hard tissue tumors;
11. Participants who are concurrently enrolled in other clinical trials and have not reached the clinical endpoints.

### Randomization and double blinding

This study used a simple random allocation. Specifically, a complete random block design was implemented via Python 3.10.10 programming to generate a randomization scheme compliant with clinical trial standards. The randomization sequence was prepared by an assistant who was not involved in treatment interventions or color assessments. Allocation details were sealed in envelopes labeled with participant numbers and group assignments. Neither the participants nor the researchers were aware of the allocation. The blinded researchers were also unaware of which group the data belonged to during data collection and analysis. The blinding was revealed after the analysis was completed. To ensure the success of the blinding, the products used by the participants in both groups, as well as the treatments and examination procedures they received, were identical in appearance.

### Baseline data analysis

Baseline data analysis, a critical step in randomized controlled trials, was conducted to provide a snapshot of participants' health status and characteristics at study entry, ensuring trial validity. In this study, baseline assessments included demographic information and medical and allergy histories collected via questionnaire, as well as comprehensive physical and oral examinations, supplemented by venous blood sampling for HCG measurement.

### Bleaching protocol

First, all patients underwent supragingival scaling. The study focused on the maxillary and mandibular anterior teeth, where participants in the treatment group used a specified toothbrush and toothpaste along with the Low-concentration Whitestrips (3% hydrogen peroxide), while the control group used a same specified toothbrush and toothpaste with regular strips (a placebo product that resembled the Low-concentration Whitestrips in appearance but lacked the active whitening ingredients). When distributing the whitening tooth strips to each participant, demonstrate the method of use and provide an instruction manual: Keep hands clean and dry when peeling off the dental strips, apply them along the gum line. Press gently to ensure they adhere to the teeth, and fold the excess parts inward. After 30 min, remove the strips, brush teeth, and rinse or brush again. Ensure that each participant is proficient in the use of the method. Participants are required to use bleaching products once every night before going to bed.

### Duration of the trial

The duration of this clinical trial was two weeks. The trial was set with three visit periods: Visit 1 (1 day), Visit 2 (3 days), and Visit 3 (14 days).

### Clinical assessment and scoring procedure

At each visit, objective and subjective evaluation methods were performed. The examiners were trained and calibrated before baseline measurements. A threshold of Kappa value >0.75 was used to qualify the examiners. During the follow-up examination, the safety assessments were performed.

### Digital spectrometer color value: primary outcomes assessment

At baseline and during each visit period, the digital shade meter (Vita Easyshade® V, Vita Zahnfabrik H. Rauter GmbH & Co. KG) was used for the digital evaluation. We selected only one maxillary central incisor for measurement, specifically the more stained one or a random selection if both were equivalent. And a colorless silicone rubber repositioning guide plate was fabricated and used to ensure repeatability of the examination site. The participant's mouth is gently held open using a mouth retractor to facilitate clear observation of the teeth, then the Vita Easyshade digital shade meter automatically displayed the CIELAB color space values of the tooth surface. The CIELAB three-dimensional color space is defined by three coordinates: L* (dark-light), a* (green-red), b* (blue-yellow). A composite color change parameter, denoted as W*, was calculated to quantify the vector distance from the overall tooth color to the white reference point. It is derived from the following formula: W*=(a*2+b*2+(L*−100)2)1/2.

### Vita shade guide color value: primary outcomes assessment

At baseline and during each visit period, a trained examiner performed the visual evaluation using 3D-Master shade guide (VITA Bleachingguide 3D-master®,Vita Zahnfabrik H.Rauter GmbH & Co. KG). To comprehensively evaluate overall smile aesthetics, we measured the color of both maxillary and mandibular anterior teeth, using the mean value as the final outcome. This shade guide consists of a series of 29 tooth-colored tabs for identifying the closest match to tooth color, with higher values indicating darker shades. The subjective color evaluations were made under the same conditions of artificial light in order to obtain accurate VITA values for manual shade matching. By comparing the average color values from the baseline and the follow-up visits, the change in tooth color for each subject is calculated. This provides a quantitative measure of the efficacy of the whitening treatment.

### Safety assessment

During the whitening process, adverse events (AEs) and serious adverse events (SAEs) based on clinical examination and participants' description were also recorded. AEs refer to transient, self-limiting, mild symptoms without sequelae, including tooth sensitivity and discomfort of mucosal and skin, etc. SAEs include recurrent, severe and self-limiting oral symptoms as well as systemic symptoms. The safety assessment was based on the clinical examination and the participants' description. The safety analysis included all participants who used the study product at least once and completed at least one follow-up visit.

### Statistical analysis

The data for both the primary and secondary outcomes generally conform to a normal distribution. Accordingly, independent sample *T*-tests were performed for inter-group comparisons, while analysis of repeated measurements were conducted within groups. Adverse events were statistically assessed using Chi-square tests. *P* value of less than 0.05 was considered to indicate statistical significance. SAS9.4 and python3.6.6 were used as statistic software.

## Results

### Baseline conditions

**Figure 1:**
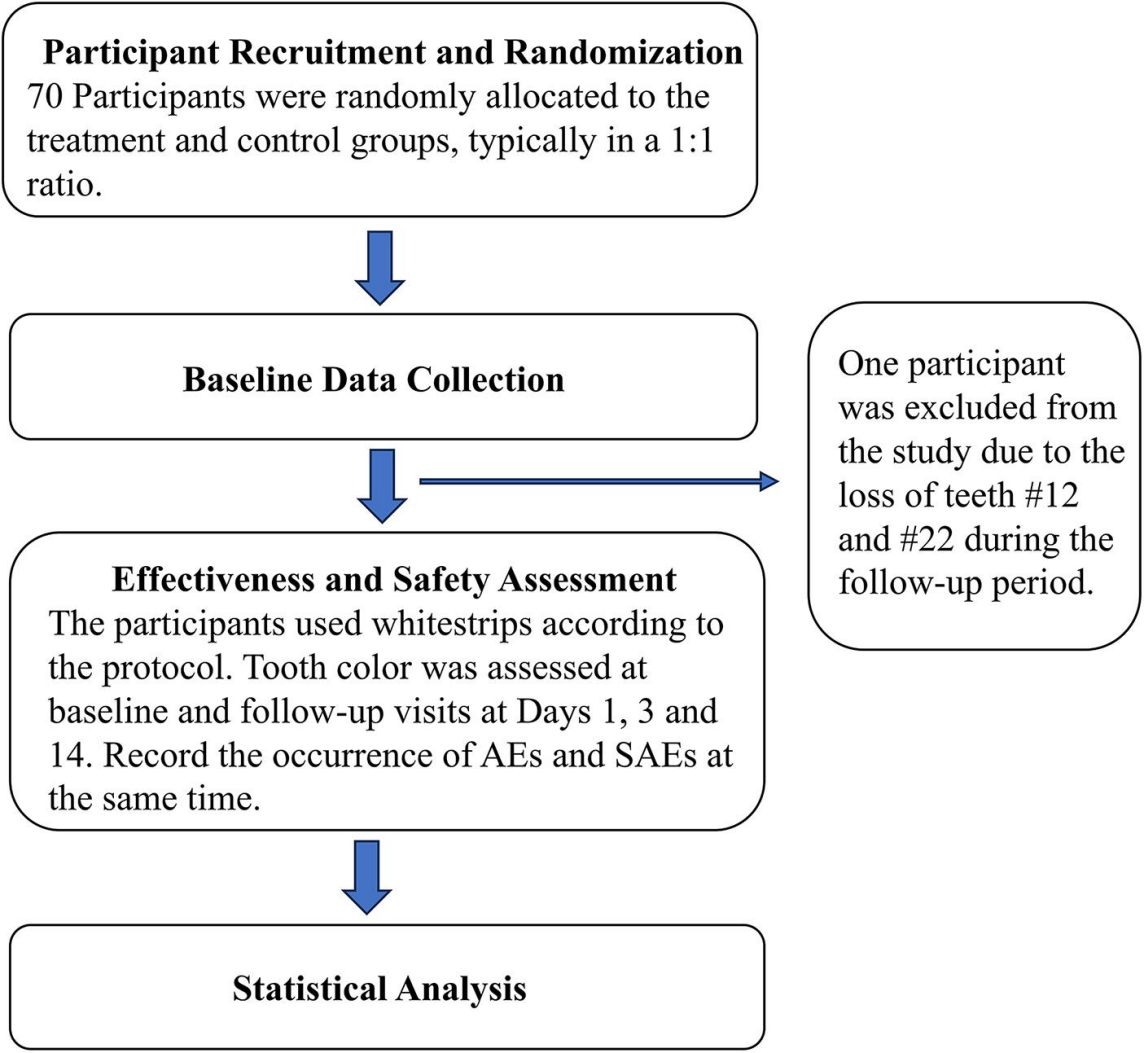
Participant flow diagram.

At the commencement of this trial, 70 individuals were enrolled as planned. Subsequently, one participant was excluded from the study due to the loss of teeth #12 and #22 during the follow-up period. In the end, 69 participants (15 males, 54 females) completed the experiment and were included to analysis. The first participant started the trial on September 22, 2023, and the last patient completed the trial on January 2, 2024 (Figure 1).

**Figure 2:**
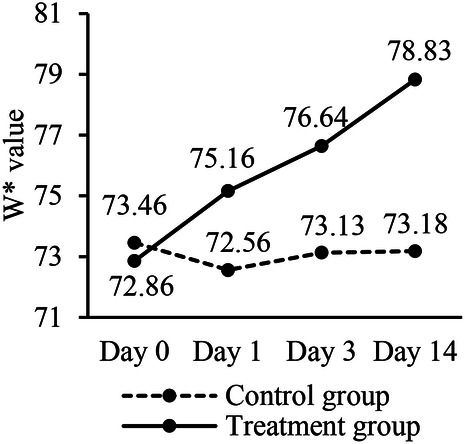
Comparison of W* values between two groups during different time points.

**Figure 3:**
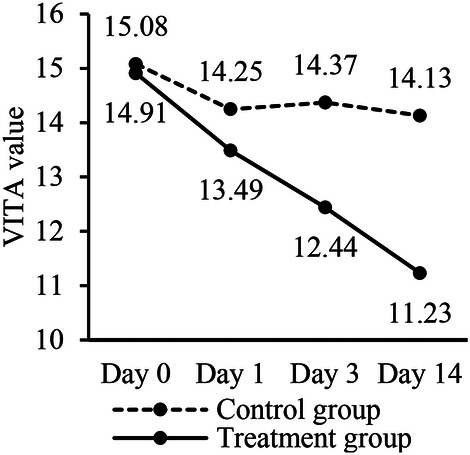
Comparison of 3D-Master VITA values between two groups during different time points.

The mean (SD) age among participants was 29 (10) years old. All participants exhibited healthy oral soft and hard tissues. And all participants showed negative HCG test results. A more detailed analysis of the baseline characteristics is provided in Table 1.

**Table 1:** Participants’ demographic background and medical information at baseline.

Variable	Statistic	Control group	Treatment group	Total	*P* value
Age (years)	Mean (SD)	29.04 (9.55)	29.42 (9.57)	29.23 (9.49)	0.995
Gender	Male	8 (11.59%)	7 (10.14%)	15 (21.74%)	0.722
	Female	26 (37.68%)	28 (40.58%)	54 (78.26%)	
Medical history	No	32 (46.38%)	35 (50.72%)	67 (97.10%)	0.460
	Yes	2 (2.90%)	0 (0.00%)	2 (2.90%)	
Allergy history	No	33 (47.83%)	34 (49.28%)	67 (97.10%)	1.000
	Yes	1 (1.45%)	1 (1.45%)	2(2.90%)	

### Digital spectrometer color value: primary outcomes assessment

The primary outcomes assessment utilized the Vita Easyshade digital shade meter, which provides a standardized method for analyzing the color of teeth. Inter-group comparison is used to assess the differences between the treatment group and the control group, whereas intra-group comparison is employed to evaluate the changes in tooth color over time within each group. Upon inter-group comparison, the W* values showed no statistically significant difference between the control group and the treatment group at baseline (*P* > 0.05). However, the W* values at Day 1, Day 3, and Day 14 demonstrated statistically significant differences (*P* < 0.05) between the two groups, with the treatment group's W* values improving more than the control group, indicating a more significant shift towards the ideal white color in the treatment group. This suggests that the whitening treatment administered to the treatment group was more effective in enhancing tooth color compared to the treatment given to the control group.

Intra group comparison showed that for the control group, the W* values at each time point (Baseline, Day 1, Day 3, and Day 14) showed no statistically significant differences (*P* > 0.05). This suggests that there was no significant change in tooth color for the control group over the course of the study. For the treatment group, however, the W* values demonstrated statistically significant differences within the group across the different time points (*P* < 0.05). The W* values at Day 1, Day 3, and Day 14 were all found to be better than at the baseline. Additionally, the W* values at Day 3 and Day 14 were both superior to those at Day 1, and the values at Day 14 showing the most significant improvement over the initial measurement (*P* < 0.05). This indicates a progressive and significant improvement in tooth color for the treatment group over the 14-day evaluation period (Table 2, Figure 2).

**Table 2:** Comparison of W* values among two groups during different time points.

Time point	Control group(*n* = 34) (Mean ± SD)	Treatment group(*n* = 35) (Mean ± SD)	*P* value
Day 0 (Baseline)	73.64 ± 4.61	72.86 ± 5.73	0.532
Day 1	72.56 ± 4.79	75.16 ± 5.58	0.041*
Day 3	73.13 ± 4.59	76.64 ± 4.81	0.003**
Day 14	73.18 ± 4.93	78.73 ± 4.25	0.000**

* *p* < 0.05.

** *p* < 0.01.

### Vita shade guide color value: primary outcomes assessment

The secondary outcomes assessment was based on the VITA 3D-Master shade guide color values (hereinafter referred to as VITA color values). Upon inter-group comparison, the VITA color values at baseline and Day 1 for the treatment group and the control group showed no statistically significant differences (*P* > 0.05). This indicates that the two groups were comparable in terms of tooth color at the outset of the study. However, at Day 3 and 14, the VITA color values between the two groups demonstrated statistically significant differences (*P* < 0.05). Specifically, the treatment group exhibited a more pronounced decrease in VITA color values compared to the control group, which implies that the teeth of the treatment group became lighter and closer to the reference white color provided by the VITA shade guide.

Intra-group comparisons of the VITA color values at different time points within each group showed the following results. For the treatment group, the VITA color values at each time point (Day 1, Day 3, Day 14) were all statistically significantly different from the baseline values (*P* < 0.05), indicating a progressive whitening effect over time. Additionally, the VITA color values at Day 3 were significantly different from those at Day 1, and the values at Day 14 were significantly different from those at Day 3 (*P* < 0.05), demonstrating a continued improvement in bleaching as the study progressed. And a color difference value higher than 2.5 is considered significant (15, 16). For the control group, the VITA color values at baseline were found to be statistically different from those at Day 1, Day 3, and Day 14 (*P* < 0.05). Specifically, the VITA color values at Day 1, 3, and 14 were all lighter compared to the baseline, but this change in tooth color is not significant. When comparing the VITA color values between Day 1 and Day 3, Day 1 and Day 14, Day 3 and Day 14 within the control group, no statistically significant differences were observed (*P* > 0.05). This implies that there was no further significant change in tooth color within the observation period in the control group (Table 3, Figure 3).

**Table 3:** Comparison of 3D-master VITA values among two groups during different time points.

Time point	Control group(*n* = 34) (Mean ± SD)	Treatment group(*n* = 35) (Mean ± SD)	*P* value
Day 0 (Baseline)	15.08 ± 3.16	14.91 ± 3.31	0.831
Day 1	14.25 ± 2.91	13.49 ± 2.96	0.288
Day 3	14.37 ± 2.94	12.44 ± 2.61	0.005**
Day 14	14.13 ± 3.01	11.23 ± 2.58	0.000**

** *p* < 0.01.

### Safety assessment

The analysis indicated that, throughout the clinical trial, a total of five AEs were reported by five participants, all of which resolved spontaneously without any lasting effects. Various details such as specific symptoms, duration, and relationship with bleaching were recorded (Table 4). The AEs that occurred during the clinical trial period in both the control group and the treatment group were compared, and no statistically significant differences were found (*P* > 0.05). Additionally, no SAEs were reported throughout the clinical trial period. This suggests that the whitening treatment was well-tolerated by the participants.

**Table 4:** All AEs during bleaching were recorded, and the incidence of AEs did not differ between the control group and the treatment group (*p* > 0.05).

Code	Group	AEs	Start time	End time	Characteristic	Degree	Relationship with bleaching
0120	Control group	Transient sensitivity of anterior teeth	2023-10-05	2023-10-05	One attack	Mild	May be relevant
0125	Control group	An ulcer about 2 mm in diameter at the lip gingiva of tooth #41	2023-10-22	2023-10-31	Persistent	Mild	May not be relevant
0138	Control group	Punctate red rashes of the mandibular skin with pruritus	2023-11-05	2023-11-13	Eight attacks	Moderate	May be relevant
0140	Treatment group	Slight gingiva numbness	2023-11-09	2023-11-09	One attack	Mild	Very likely to be relevant
0153	Treatment group	Blistering on the upper and lower right lips	2023-12-27	2024-01-05	Persistent	Mild	May not be relevant

## Discussion

In this study, both the primary and secondary outcomes were used to assess the same clinical variable, namely tooth color change, with the former providing an objective digital measurement and the latter reflecting subjective visual evaluation. During the treatment period, the treatment group's W* values (primary outcomes) increased from 72.86 to 78.73, and the VITA 3D-Master shade guide color values (secondary outcomes) decreased from 14.91 to 11.23, corresponding to a mean shade change of approximately 3.68 shade guide units (ΔSGU). These findings indicate a significant change in tooth color as assessed by both objective digital analysis and subjective visual evaluation. Notably, previous studies employing the same VITA 3D-Master shade guide system have reported varying magnitudes of whitening for different HP–based products. For example, Chemin et al. (17) reported a *Δ*SGU of approximately 5.3 after two weeks of at-home bleaching with 4% HP, whereas Carneiro et al. (12) observed a shade change of approximately 2.6 ΔSGU following in-office bleaching with 6% HP. The observed differences in whitening outcomes may be attributable to variations in patient compliance, application techniques, and clinical settings. Nevertheless, all studies consistently demonstrated the whitening efficacy of HP, supporting its effectiveness across different concentrations.

Extrinsic tooth discoloration refers to changes in tooth color that are not caused by alterations within the tooth's internal tissue structure. It is typically a result of pigment deposition. The main causes of extrinsic staining include dietary factors, such as long-term intake of foods and beverages containing pigments (18). Smoking is also contributory to tooth discoloration, with toxins of tobacco smoke accumulating in a similar way (19). In addition, certain medications, such as long-term gargling with chlorhexidine may also form coloring on the tooth surface (20).

This type of discoloration can be removed through proper cleaning and whitening methods. Clinical studies on tooth whitening with HP have garnered attention, and their efficacy and safety have been reported in various case studies and clinical trials (3). The fundamental principle of tooth whitening relies on the redox reaction of HP. When HP is oxidized, it releases oxygen and forms superoxide radicals (HO2−). These radicals quickly penetrate the tooth surface and dentin tubules, breaking down pigments that are attached to the tooth surface and deep layers (21). HO2− has a strong oxidizing effect, which can degrade pigment molecules from large to small molecules, facilitating their dispersion outside the teeth. Furthermore, HO2− can react with pigment substances, converting organic pigments such as carbon ring structures into non-pigmented hydrophilic structures. The compounds that have undergone this transformation are replaced with non-pigmented simple compounds, while the remaining molecules are broken down into safe and harmless water molecules, which are then released (22).

While HP is an effective bleaching agent, awareness of its potential risks, particularly at high concentrations, is important. The most common adverse reaction during the bleaching process is tooth sensitivity. HP not only interacts with pigments but also permeates into normal tissues, where it undergoes oxidative reactions, leading to the loss of carbonates and proteins in the enamel and dentin. These reactions also cause alterations in hydroxyapatite and damage to the tooth surface, ultimately resulting in tooth sensitivity (23). The redox reaction of HP can also cause tooth demineralization, altering the microstructure of the hard tissues as well as the biomechanical properties of the surface, leading to a decrease in the microhardness of the teeth (24, 25). Furthermore, due to its corrosive nature, HP can cause chemical burns to soft tissues if the whitening agent comes into direct contact with the oral mucosa, with temporary irritation to the gingiva being particularly common (26). HP has also been shown to have cytotoxic effects. HP can spread through the dental structure because of its low molecular weight, leading to oxidative stress in the pulp cells and the subsequent inflammatory reactions. When used in excessively high concentrations, it may even provoke pulpitis (27, 28).

Therefore, recent trends in clinical research have increasingly emphasized safety considerations in tooth whitening treatments, with greater caution regarding the clinical use of high-concentration HP (29, 30). Many studies have focused on the whitening effects of low concentrations HP because they are more friendly to oral tissues and also effective (6, 12, 31). However, the commonly used whitening products with a concentration of more than 6% HP still have light cytotoxicity (10–13). In order to find effective and safer bleaching products, the Low-concentration Whitestrips used in this trial only contains 3% HP.

In order to provide a comprehensive view, we have employed both primary and secondary outcomes to assess the effectiveness of Low-concentration Whitestrips from both subjective and objective perspectives. The primary outcomes were assessed using the Vita Easyshade digital shade guide, which provides colorimetric values for an objective evaluation of tooth color. Advances in digital camera technology and image analysis software have provided the basis for a more accurate assessment of tooth color (32). And digital image analysis has been shown to have high *in vivo* and *in vitro* reproducibility. The Vita Easyshade digital shade meter can capture the color of the tooth and translate it into colorimetric values within the CIELAB color spaces. Use of peroxide-based whitening intensives leads to measurable differences in all three CIELAB parameters, specifically increased lightness (+L*), decreased redness (−a*) and decreased yellowness (−b*) (33). Additionally, a composite color change parameter, W*, is calculated to measure the overall color change relative to an abstract white color, providing a comprehensive assessment of the whitening effect. The higher the W* value, the whiter the teeth.

Inter-group analysis revealed that on both Day 3 and Day 14, the W* values in the treatment group were higher than those in the control group. This indicates the Low-concentration Whitestrips demonstrated greater whitening efficacy compared to the placebo. Intra-group analysis showed no change in tooth whiteness for the control group. In contrast, the treatment group exhibited an increase in whitening effectiveness over time. This progressive improvement suggests that the prolonged use of the Low-concentration Whitestrips leads to increasingly better results, highlighting the potential benefits of extended treatment duration with the whitening product. The results of this experiment are consistent with those reported by Pinto et al (34) and Chen et al. (35), supporting the effectiveness of low-concentration whitening formulations. It is worth noting that the control group was darker on Day 1 compared to the baseline period, which may may be caused by errors in operation or environment, and has no statistical significance.

Although the primary outcomes provide a more objective assessment of the whitening effects, the perception of color is not solely based on the physics of optics. It also involves physiological and psychological factors. The moisture level can affect the glossiness and reflectivity of the tooth surface, which may affect the perception of the examiner. The intensity and color temperature of light also play a crucial role in how tooth color is perceived by the human eye (36). Additionally, the color contrast effect can alter the perception of tooth color. When a tooth is observed adjacent to the gums, skin and other tooth, a contrast effect may occur, influencing the perceived color (37). As a result, computerized analysis may not fully replicate the human eye's ability to perceive color under complex lighting conditions.

Therefore, this trial also incorporates the secondary outcomes based on the VITA 3D-Master shade guide to subjectively evaluate the effects of the whitening strips, which is a widely recognized tool for shade matching in dentistry (38). We utilized the VITA shade guide color values for comparison, with lower values indicating whiter teeth. The secondary outcomes analysis yielded results consistent with the primary outcomes, suggesting that, from a subjective standpoint, the whitening effect of Low-concentration Whitestrips was better than that of placebo, and the longer the effect was better. Generally, a color difference value higher than 2.5 is considered significant (15, 16), which means there is a significant visible bleaching effect on the Day 14 compared to the Baseline (14.91–11.23). Slightly different from the primary outcomes, the intra-group analysis of the secondary outcomes showed that the teeth whitening degree at Day 1, Day 3, and Day 14 was higher than that of the baseline period in the control group, while there was no differences in the whitening degree at the visit periods. The lack of further significant changes in the control group after the baseline could be attributed to the natural variation or minor fluctuations in tooth color that do not indicate a true whitening effect. By comparison, the consistent statistically significant improvements in the treatment group at each time point reinforce the efficacy of the whitening treatment.

Additionally, the safety of the Low-concentration Whitestrips was evaluated throughout the trial. A total of five adverse events (AEs) were reported, with three occurring in the control group and two in the treatment group. There is no statistical difference between the two groups. The primary adverse reactions observed were tooth sensitivity and mucosal discomfort. These reactions were mild, and the symptoms resolved spontaneously without any treatment, leaving no sequelae. No serious adverse events (SAEs) were reported.

However, some limitations need to be described. Although this study confirms the effectiveness of the Low-concentration Whitestrips, providing important preliminary evidence for a milder and safer treatment, we acknowledge that the lack of data on long-term color stability is a clear limitation that awaits clarification in future longitudinal studies. And despite training participants in proper use of the whitening strips, home application could not be strictly supervised, potentially introducing variability in adherence and technique that may have influenced bleaching outcomes. Furthermore, the sample size was not based on a formal power calculation. This study was designed as an exploratory trial due to the absence of prior data. Therefore, while the observed effects were significant, the study should be considered preliminary. The data from this trial should serve as a basis for power calculations in future, large-scale confirmatory studies.

In conclusion, our research demonstrates that Low-concentration Whitestrips with 3% HP not only deliver a satisfactory bleaching effect but also enhance their efficacy over time while maintaining a high degree of safety. The findings from both the primary and secondary outcomes assessments reinforce each other, providing a comprehensive view of the whitening strips' performance, enhancing the internal validity. Considering the strong representativeness of the subjects we recruited, the results can be applied to healthy adults aged 18–55 with extrinsic discoloration on their incisors, thus ensuring the external validity.

## Conclusions

In this randomized controlled trial, low-concentration whitestrips containing 3% hydrogen peroxide demonstrated significant efficacy for treating extrinsic tooth discoloration. The treatment was well-tolerated, with the incidence of adverse events being statistically comparable to the placebo group. These findings provide robust clinical evidence for dentists and patients, establishing these whitestrips as a reliable at-home whitening option.

## Data Availability

The original contributions presented in the study are included in the article/Supplementary Material, further inquiries can be directed to the corresponding author.
